# Informing the Design of Large-Scale Food Fortification Programs with Secondary Data: Pilot Results from Nigeria and Zambia

**DOI:** 10.1016/j.cdnut.2024.104522

**Published:** 2024-12-10

**Authors:** Katherine P Adams, Emmanuel A Gyimah, Svenja M Jungjohann, Jacqueline L Hems, Musonda J Mofu, Olufolakemi Mercy Anjorin, Jennifer Yourkavitch, Silvia Alayón, Heather Danton, Ingrid Weiss, Omar Dary, Monica B Woldt

**Affiliations:** 1 Department of Nutrition, Institute for Global Nutrition, University of California, Davis, California, United States; 2 Global Alliance for Improved Nutrition, Geneva, Switzerland; 3 Independent Consultant, Gloucestershire, United Kingdom; 4 Independent Consultant, Lusaka, Zambia; 5 Independent Consultant, Abuja, Nigeria; 6 USAID Advancing Nutrition, Arlington, Virginia, United States; 7 Results for Development, Washington, DC, United States; 8 Save the Children, Washington, DC, United States; 9 Bureau for Resilience, Environment, and Food Security, USAID, Washington, DC, United States; 10 Bureau for Global Health, USAID, Washington, DC, United States; 11 Helen Keller International, Washington, DC, United States

**Keywords:** Nigeria, Zambia, large-scale food fortification, micronutrients, dietary modeling, market data, Cost of the Diet

## Abstract

**Background:**

Large-scale food fortification (LSFF) can improve micronutrient intake, but low-income and middle-income countries often lack resources to collect data for LSFF decision making. We designed a methodology using existing data and methods to inform LSFF programming.

**Objectives:**

This study aimed to pilot a methodology using existing diet, market, and diet cost data and assess its implementation feasibility and usefulness for LSFF decision making.

**Methods:**

We used household food consumption/availability data from Nigeria and Zambia to assess dietary micronutrient inadequacy and model contributions of LSFF. Market survey (Nigeria) and agrifood information system (Zambia) data were used to estimate availability of fortifiable foods. We used Cost of the Diet software to estimate affordability of an adequate diet in Zambia. We engaged country stakeholders to determine modeling parameters and assess methodology implementation.

**Results:**

The analyses took ∼6 mo and cost ∼150,000 USD (Nigeria) and ∼250,000 USD (Zambia). Results showed diets were inadequate to meet vitamin A, folate, and zinc requirements of 32%–67% of households in Nigeria and 51%–83% of households in Zambia. Modeling existing LSFF in Nigeria demonstrated improved micronutrient adequacy of diets, with further improvements possible with rice fortification. In Zambia, existing vitamin A–fortified sugar marginally reduced inadequacy. Introducing mandatory wheat flour and/or maize flour fortification could reduce folate and zinc inadequacies. The market assessment demonstrated widespread availability of fortifiable edible oil, sugar, and wheat flour, but not fortifiable maize flour. The cost of the diet in Zambia with LSFF was similar to the cost without LSFF. Stakeholders found the methodology’s components together generated useful, timely evidence for LSFF programming.

**Conclusions:**

Our methodology showed opportunities for improving the micronutrient adequacy of diets in Nigeria and Zambia through LSFF. The methodology generated evidence that stakeholders agreed can support LSFF planning. Investments and strategies are needed to strengthen capacity to conduct the assessments, reduce costs, and streamline methods.

## Introduction

Micronutrient deficiencies affect an estimated 372 million preschool-aged children and 1.2 billion nonpregnant women of reproductive age worldwide [1]. Single or multiple micronutrient deficiencies can negatively affect maternal health and child growth and cognitive development, morbidity, and mortality, and, in the long-term, achievement in school and work and overall economic development [[2], [3], [4]]. Micronutrient malnutrition can have various causes, including inadequate intake from dietary sources [4]. Globally, micronutrients with the highest estimated prevalence of inadequate intake are calcium, iron, vitamin A, folate, zinc, riboflavin, and vitamin B-12, particularly in low-income and middle-income countries (LMICs) [5]. Evidence shows that large-scale food fortification (LSFF), the addition of vitamins and minerals to commonly consumed staple foods, condiments, and food ingredients during industrial processing, has contributed to a reduction in the estimated prevalence of inadequate micronutrient intake in the majority of low-income regions of the world [5]. When appropriately implemented, LSFF is a safe and effective strategy to improve micronutrient intake and decrease risk of deficiencies [6,7]. Despite LSFF programs reaching an increasing number of people in LMICs over the past 2 decades, its potential has not been reached in terms of adoption and effective implementation [8]. There are critical gaps in LSFF program design and implementation, including fortification of food vehicles with low coverage among target populations and inadequate monitoring and enforcement of fortification standards. These gaps are due in part to inadequate use of existing data, as well as lack of clear methods for analyzing data and interpreting and using the results for needs assessment and evidence-based decision making to design or redesign LSFF programs [[8], [9], [10]].

To fill this gap, we designed a methodology that uses existing data, analyzed using existing methods, to assess diets, markets, and the cost and affordability of an adequate diet to inform the design of LSFF programs [[11], [12], [13]]. We designed the methodology for use by United States Agency for International Development (USAID) Missions and public and private sector partners. We first conducted a literature review to understand and catalog the data sources and methods used to inform various aspects of LSFF program design [13]. The review provided insights to prioritize data sources and methods based on their strengths and limitations. In consultation with experts, we then synthesized these existing methods and data sources to develop a comprehensive methodology, including a decision tree to guide the selection of existing data for the analysis, which are presented in an Operational Overview [12] and Methods Guide [11]. We piloted the methodology in Nigeria and Zambia, based on expressed interest by the USAID Mission in each country and government interest in LSFF.

The methodology laid out in the Methods Guide [11] recommends first conducting a needs assessment to identify micronutrients that are insufficient in diets. The needs assessment is followed by a LSFF design/redesign step that includes estimating consumption of fortifiable foods, modeling the contributions of LSFF to meeting micronutrient requirements (as well as risk of high intakes), and determining the market availability of fortifiable and fortified foods. The final step, a cost of the diet (CotD) analysis, is optional and intended for use in advocacy to demonstrate the potential impact of LSFF on the affordability and accessibility of a micronutrient adequate diet compared with a diet without fortification. In this article, we present selected results of piloting this methodology in Nigeria and Zambia to demonstrate the types of evidence that practitioners can generate. As part of the piloting process, we also assessed the feasibility of implementing, and identified needed improvements to, the methodology. A second objective of this article was therefore to present indicators of feasibility (including duration and cost) of implementing the methodology and suggested improvements to the methodology that were identified during piloting. Finally, during piloting, we engaged stakeholders from Nigeria and Zambia to help determine modeling parameters and assess the usefulness of the results of the methodology. As such, the final objective of this article was to describe the stakeholder engagement process and stakeholder perceptions of the potential usefulness of the methodology for LSFF decision making. Taken together, the results and information presented in this article can help practitioners and countries assess the potential for using the methodology to generate data-driven evidence to support LSFF planning in their own contexts.

## Methods

### Needs assessment

There are several potential sources of data to estimate food consumption and the prevalence of micronutrient inadequacies without LSFF or other micronutrient interventions. Nationally representative 24-h dietary recall data are the preferred source, followed by semiquantitative food frequency data and household consumption and expenditure survey (HCES) data [12,14]. In both Nigeria and Zambia, at the time of the piloting, the best publicly available data were from HCESs. As such, we used data from the 2018–2019 Nigeria Living Standards Survey (NLSS) [15] and the 2015 Zambia Living Conditions Monitoring Survey (LCMS) [16].

Although both the NLSS and the Zambia LCMS were categorized as household consumption and expenditure surveys, the methods used to collect data on household food consumption and/or acquisition varied substantially between the 2 surveys. The food consumption module of the NLSS collected data from the household respondent on total quantities of each of 99 prespecified foods consumed by household members in the 7 d preceding the survey, including the source of food consumed (purchases, home production, or gifts or other sources). The NLSS data set also included conversion factors to enable the conversion of quantities of food reported in nonstandard units (e.g. piles, bowls, sacks) to grams. In Zambia, the food module of the LCMS collected data from the household respondent on the quantities of each of 129 foods that were acquired (rather than consumed) by the household via purchases or received without payment (gifts, food for work, or relief food) as well as foods consumed by the household from own production. The recall period for maize grain, breakfast mealie meal, roller meal, hammer mealie meal, pounded maize meal, salt, spices, and cooking oil was the 4 wk preceding the survey and for all other food items the 2 wk preceding the survey.

Neither the data nor other Zambia-specific sources included a comprehensive set of conversion factors, so, where possible, we converted quantities of food acquired and consumed to grams using available country-specific conversion factors and conversion factors from the Malawi Fifth Integrated Household Survey [17]. We did not have conversion factors for ∼25% of reported quantities of food acquired, so we imputed price-per-gram estimates for each food item in order to convert reported quantities acquired to grams. Specifically, for each food in the food list, based on observations of quantities acquired with conversion factors, we divided the reported price paid by the total quantity (in grams) to estimate an urban and rural residence-specific median price-per-gram. Then, for the 25% of observations without conversion factors, we used the median price-per-gram (specific to the food item and urban compared with rural residence) and the reported amount paid to estimate the quantity acquired in grams.

In both countries, we adjusted quantities of food for the edible portion and yield factor from cooking where relevant. We identified and managed potentially implausible quantities of food consumed/acquired based on apparent food consumption per day per adult female equivalent (AFE, described later). Specifically, for each food item, we calculated the zone-specific (Nigeria) or province-specific (Zambia) 95th percentile of apparent consumption per day per AFE among consumers; quantities apparently consumed above the 95th percentile were replaced with the 95th percentile of apparent consumption [18]. In total, household food consumption observations were available for 22,116 households in the NLSS data set and 12,234 households in the Zambia LCMS data set. All estimates account for survey weights included in the NLSS and LCMS data sets.

Once apparent household consumption or acquisition of all foods were converted to grams, based on input from country collaborators and local stakeholder groups, we matched each food with a food composition table (FCT) entry (or a weighted average of several entries in the case of aggregate food items) to estimate the nutrient composition. For Nigeria, most foods were matched to the West African FCT [19] and, where appropriate matches were not available, supplemented with entries from the Nutrition Coordinating Center Nutrient Database for Standard Reference [20] or the Malawian FCT [21]. Items in the Zambia LCMS were primarily matched with the Zambia FCT [22] and supplemented with entries from the Malawi FCT. Note that the Nigerian food list included palm oil as a food item without distinguishing between types of palm oil. The list also included groundnut oil. Based on input from the Nigeria stakeholder group that reported consumption of palm oil in Nigeria virtually exclusively refers to red palm oil, we assumed that all reported consumption of palm oil was unrefined red palm oil. The Nigeria stakeholder group also reported that refined oils in Nigeria are generically considered vegetable oils. Therefore, for each geopolitical zone in Nigeria we disaggregated reported groundnut oil consumption into *1*) groundnut oil, which we assumed to be refined at small-scale and hence not fortifiable, and *2*) refined, fortifiable palm and soybean oils. Supplemental Table 1 presents zone-specific proportions.

We estimated daily apparent household intake of vitamin A, folate, and zinc per AFE (note that the results presented in this article focus on these micronutrients as examples, but the methodology and piloting also cover thiamin, riboflavin, niacin, vitamin B-6, vitamin B-12, and iron). The number of AFEs in each household was determined based on each household member’s age-specific and sex-specific energy requirements relative to the energy requirements of a nonpregnant, nonlactating adult female aged 18–29.9 years [23,24]. We selected adult females as the reference group because their food consumption is expected to be approximately the average in the household, and their micronutrient requirements are generally high relative to other household members [24]. Because the surveys did not collect pregnancy or breastfeeding status, we did not account for higher energy requirements during pregnancy, and we made several assumptions to adjust for energy requirements met via breastmilk among children younger than 2 y and the increased energy requirements during lactation. The Supplemental Methods detail methods and assumptions for determining energy requirements. We calculated an AFE weight for each household member by dividing the energy requirement of each household member by the energy requirement of the reference female. Then, we divided daily apparent household intake of each micronutrient by the sum of all AFEs in the household to estimate daily apparent intake per AFE. We use “apparent” to emphasize the assumptions inherent in using household-level data to assess micronutrient adequacy, including that all food acquired during the recall period was consumed during this window (Zambia). We also assumed that household members distributed food in proportion to energy requirements [25].

To assess the adequacy of the household diet for meeting the micronutrient requirements of the reference adult female, we compared daily apparent micronutrient intake per AFE to the estimated average requirement for the reference adult female. Estimated average requirements were based on harmonized average requirements (H-ARs) [26]. We selected zinc requirements corresponding to unrefined diets based on apparent phytate intake of ∼1200 mg/day per AFE in Nigeria and for Zambia based on recommendations from the local stakeholder group on usual phytate intake (the phytic acid content of foods was not included in the Zambia FCT; personal communication with Zambia stakeholder group during online meeting, May, 2023). Using the cutpoint method, households with apparent micronutrient intake per AFE below the H-AR were categorized as inadequate. These estimates do not account for supplementation or other micronutrient interventions (e.g. biofortification), as data on these interventions were not collected as part of the household surveys.

### LSFF design/redesign: consumption of fortifiable foods and the contribution of LSFF

We estimated apparent consumption of 2 types of potentially fortifiable food vehicles: food vehicles that *1*) currently have national mandatory or voluntary fortification standards regulating their fortification with vitamin A, folate, and/or zinc (refined oil, sugar, wheat flour, and maize flour in Nigeria; and sugar, wheat flour, and maize flour in Zambia) and *b*) country stakeholder groups identified as of interest for fortification (rice in Nigeria, and refined oil and rice in Zambia). We estimated apparent consumption of these food vehicles by dividing total household daily apparent consumption from purchases by the number of AFEs in the household. Where relevant, quantities of daily apparent consumption of the food vehicle included reported consumption of the food vehicle itself as well as food vehicle equivalents of processed foods containing the food vehicle (e.g. wheat flour in bread and refined oil in fritters) estimated based on recipes. We calculated both the proportion of households consuming the food vehicle as well as the median apparent quantity consumed per AFE; the latter was based on quantities consumed among consumers only.

We modeled the contribution of LSFF for meeting the dietary micronutrient requirements of the reference adult female under several scenarios. For food vehicles with existing mandatory fortification standards, we modeled a status quo scenario and an improved compliance scenario (TABLE 1, TABLE 2) [[29], [44], [45]]. The status quo scenario reflects estimates of the current situation in each country in terms of *1*) the proportion of the food vehicle that is fortifiable, *2*) the proportion of the fortifiable food vehicle currently fortified to any extent, *3*) the average fortification level relative to the standard, and *4*) expected micronutrient losses from point of fortification to homes. These parameter values were based on available data or assumptions supported by stakeholder group feedback (see notes beneath TABLE 1, TABLE 2 for modeled micronutrient losses and the sources of those parameters). In practice, we operationalized these parameters in the modeling by calculating an overall average fortification level, which was adjusted down by the percent of the food vehicle that is fortifiable, the proportion of the food vehicle that is fortified to any extent, the average fortification level among the fortified vehicle as a percent of the standard, and expected losses.

**TABLE 1:** Modeling parameters by food vehicle—Nigeria.

Description of parameter	Status quo		Improved/realistic compliance	
	Value	Source	Value	Source
Refined oil (excluding groundnut oil)				
Fortifiable	80%^1^	Estimated based on data collected by TechnoServe^2^	80%	Estimated based on data collected by TechnoServe^2^
Fortifiable food vehicle fortified to any extent	31%	NFCMS 2021 Final Report [29]	80%	Modeling assumption
Average fortification level among fortified food vehicle as a percentage of the standard^3^	62%	NFCMS 2021 Final Report [29]	100%	Modeling assumption
Sugar				
Fortifiable	100%	Assumption	100%	Assumption
Fortifiable food vehicle fortified to any extent	74%	NFCMS 2021 Final Report [29]	80%	Modeling assumption
Average fortification level among fortified food vehicle as a percentage of the standard^3^	59%	NFCMS 2021 Final Report [29]	100%	Modeling assumption
Wheat flour				
Fortifiable	96%	Global Fortification Data Exchange [44]	96%	Global Fortification Data Exchange [44]
Fortifiable food vehicle fortified to any extent	26% (vitamin A), 100% (folic acid and zinc)	NFCMS 2021 Final Report [29] and expert opinion	80% (vitamin A), 100% (folic acid and zinc)	Modeling assumption
Average fortification level among fortified food vehicle as a percentage of the standard^3^	80% (vitamin A), 74% (folic acid and zinc)	NFCMS 2021 Final Report [29] and expert opinion	80% (vitamin A), 100% (folic acid and zinc)	Modeling assumption
Maize flour				
Fortifiable	20%	Global Fortification Data Exchange [44]	20%	Global Fortification Data Exchange [44]
Fortifiable food vehicle fortified to any extent	26% (vitamin A), 100% (folic acid and zinc)	Expert opinion (assumed similar to wheat flour)	80% (vitamin A), 100% (folic acid and zinc)	Modeling assumption
Average fortification level among fortified food vehicle as a percent of the standard^3^	80% (vitamin A), 74% (folic acid and zinc)	Expert opinion (assumed similar to wheat flour)	80% (vitamin A), 100% (folic acid and zinc)	Modeling assumption
Rice				
Fortifiable	NA	NA	30%	Estimated based on data collected by TechnoServe [45]
Fortifiable food vehicle fortified to any extent	NA	NA	80%	Modeling assumption
Average fortification level among fortified food vehicle as a percentage of the standard^3^	NA	NA	100%	Modeling assumption

Abbreviation: NFCMS, National Food Consumption and Micronutrient Survey.

1 The estimate of fortifiable refined oil excludes groundnut oil, which is refined by small-scale industries in Nigeria and is not feasible for fortification.

2 Ayodele Tella, TechnoServe, personal communication, Nigeria, 20 April, 2023. Based on data collected by TechnoServe on the volume of edible oil processed by large producers.

3 Calculated relative to the standard and adjusted for expected micronutrient losses/degradation of 30% of vitamin A added to oil and sugar, 50% of vitamin A added to wheat flour or maize flour, and 20% of folic acid added to wheat flour or maize flour, based on expert opinion.

**TABLE 2:** Modeling parameters by food vehicle—Zambia.

Description of parameter	Status quo		Improved/realistic compliance	
	Value (%)	Source	Value (%)	Source
Refined oil				
Fortifiable	NA	NA	70	Estimate based on data collected by TechnoServe [45]
Fortifiable food vehicle fortified to any extent	NA	NA	80	Modeling assumption
Average fortification level among fortified food vehicle as a percentage of the standard^1^	NA	NA	100	Modeling assumption
Sugar				
Fortifiable	90	Estimated based on data collected by TechnoServe [45]	90	Estimated based on data collected by TechnoServe [45]
Fortifiable food vehicle fortified to any extent	100	Zambia FCMS 2020–2021 Preliminary Findings (2022)^2^	100	Modeling assumption
Average fortification level among fortified food vehicle as a percentage of the standard^1^	38	Zambia FCMS 2020–2021 Preliminary Findings (2022)^2^	100	Modeling assumption
Wheat flour				
Fortifiable	NA	NA	100	Country stakeholder group input, May 2023
Fortifiable food vehicle fortified to any extent	NA	NA	80	Modeling assumption
Average fortification level among fortified food vehicle as a percentage of the standard^1^	NA	NA	100	Modeling assumption
Maize flour				
Fortifiable	NA	NA	35	Global Fortification Data Exchange [44]
Fortifiable food vehicle fortified to any extent	NA	NA	80	Modeling assumption
Average fortification level among fortified food vehicle as a percentage of the standard^1^	NA	NA	100	Modeling assumption
Rice				
Fortifiable	NA	NA	68	Global Fortification Data Exchange [44]
Fortifiable food vehicle fortified to any extent	NA	NA	80	Modeling assumption
Average fortification level among fortified food vehicle as a percentage of the standard^1^	NA	NA	100	Modeling assumption

Abbreviation: FCMS, Food Consumption and Micronutrient Survey.

1 Calculated relative to the standard and adjusted for expected micronutrient losses/degradation of 30% of vitamin A added to oil and sugar, 50% of vitamin A added to wheat flour or maize flour, and 20% of folic acid added to wheat flour or maize flour, based on expert opinion.

2 Musonda Mofu, Independent Consultant, personal communication, Zambia, 30 May, 2023.

The improved compliance scenario reflects a realistic, although not yet fully achieved, situation in which 80% of the fortifiable food vehicle is fortified to the national standard (i.e. 100% of the standard after adjusting for expected losses from point of fortification to households). For food vehicles without an existing mandatory national standard, we modeled a realistic compliance scenario assuming 80% of the food vehicle is fortified to the hypothetical national standard. Here too, we adjusted down the overall average fortification level to operationalize these assumptions in the modeling.

Modeled fortification levels (Table 3) [[30], [46], [47], [48]] were adjusted to reflect these scenarios, and then, we multiplied the adjusted fortification levels by total daily apparent consumption per AFE of the food vehicle to estimate the additional micronutrients contributed to the household diet via LSFF. We then added this value to daily apparent intake per AFE without LSFF and compared the sum with the H-AR to estimate the prevalence of inadequate intake per AFE with LSFF. For vitamin A (in the form of preformed retinol) and folic acid, we also compared daily apparent intake per AFE with LSFF to the harmonized tolerable upper intake level (H-UL) for the reference adult female [26] to estimate risk of high intakes with LSFF. We converted folic acid from fortification to dietary folate equivalents by dividing the folic acid content by 0.6 [27].

**TABLE 3:** Mandated and hypothetical fortification levels.

Food vehicle	Vitamin A	Folic acid	Zinc	Source
Nigeria				
Mandated fortification level (mg/kg)				
Refined oil	6			Federal Government of Nigeria [30]
Sugar	7.5			
Wheat flour	2	2.6	50	
Maize flour	2	2.6	50	
Hypothetical fortification level (mg/kg)				
Rice	3.12	2.54	72	WFP [46]
Zambia				
Mandated fortification level (mg/kg)				
Sugar	15			SI 155^1^
Hypothetical fortification level (mg/kg)				
Refined oil	25			SADC [47]
Wheat flour	5.9	5	95	WHO [48]
Maize flour	1.5	1.3	40	WHO [48]
Rice	3.12	2.54	72	WFP [46]

Abbreviations: SADC, Southern African Development Community; WFP, World Food Program.

1 Zambia Statutory Instrument (SI) No. 155, enacted on 18 December, 1998, sets 10 mg/kg as the legal minimum level of vitamin A in sugar at the point of sale. To ensure this level, and considering an estimated 30% of losses from the point of fortification to homes, the necessary level of fortification at the factory is ∼15 mg/kg.

We calculated the prevalence of inadequate micronutrient intake per day per AFE without and with LSFF, as well as median apparent consumption of potentially fortifiable food vehicles, at the national-level, by urban and rural residence, and by quintiles of household socioeconomic strata (SES) in urban and rural areas. Estimates of household SES were based on total annual household expenditures per capita and divided into 5 even quintiles in rural areas and in urban areas. We did not test for statistical differences in our comparisons of the prevalence of micronutrient inadequacy and consumption of potentially fortifiable food vehicles across subpopulations and food vehicles.

### LSFF design/redesign: market availability of fortifiable foods

For the Nigeria market analysis, we used existing data from a 2021 cross-sectional market assessment conducted by the Global Alliance for Improved Nutrition (GAIN) and Ipsos Nigeria, a market research firm (Global Alliance for Improved Nutrition, Unpublished report, 2021). GAIN and Ipsos Nigeria collected data from a representative sample of 3000 retailers that allowed for analysis nationally, by geopolitical zone, and by vendor type (bakery, grocery/retail shop, kiosk, market stall, supermarket, and wholesaler). They collected data in 57 cities and towns, 19 each in urban, semiurban, and rural locations, using a population proportional to size multistage sampling approach and the most recent Nigeria National Bureau of Statistics Population and Housing Census data available at the time. In each market place, vendors were randomly selected. The proportion of vendor types included 2% bakery, 38% grocery/retail shop, 16% kiosk, 7% market stall, 11% supermarket, and 26% wholesaler. For the Zambia market analysis, we used FAO and Zambian Food Balance Sheets, available research reports on the food industry, and websites and social media of companies and online stores for information on national-level domestic supply, local producers, and their annual production and percent volume share (Supplemental Table 2). For both countries, we aimed to estimate the proportion of the product volume of the food vehicle that is fortifiable. In Nigeria, we estimated the percentage of the food vehicle that is fortifiable as the proportion of the total supply volume, both domestic and imported, from large producers/suppliers, considering the proportion of processors and brands found in the retail market during the GAIN and Ipsos Nigeria survey. In Zambia, we calculated the proportion of the production volume that is fortifiable among local processors and brands identified from the available sources. For most of the food vehicles, local Zambian production provided the majority of the supply. We used USAID criteria to define large-scale producers in low-income countries [51] (Supplemental Table 3). We crosschecked sources and standardized unit conversions. We assumed that *1*) domestic supply = (local production) − (exports) + (imports) − (stock variation) as used in FAO Food Balance Sheets; and *2*) annual production capacities of food producers were proportionate to the actual annual production volume. We analyzed the Zambia market data nationally only, given the available data.

### Cost and affordability of the diet

We used the CotD software [28] to assess the cost and affordability of an adequate diet without and with LSFF nationally and by province in Zambia. We used population expenditure data from the 2015 LCMS to compare food expenditures with the cost of a micronutrient adequate diet. We estimated the proportion of the population that would not be able to afford the micronutrient adequate diet without and with fortified foods. We used Zambian consumer price index (CPI) data to adjust the 2015 LCMS food expenditures to February 2023 prices. This inflated the price of all foods equally and therefore did not account for differential changes in prices across different types of foods.

We estimated the cost and affordability of a nutritious diet adequate in micronutrients without and with fortified foods for a 5-person household including an adult man, an adult breastfeeding woman, an adolescent girl, a school-aged child, and a breastfed child aged 6–23 mo. This is the same modeled household used in the World Food Program (WFP) 2021 Fill the Nutrient Gap CotD analysis [38]. It should be noted that the nutritionally adequate diets created are hypothetical and not necessarily reflective of what a typical household might consume daily. We selected parameter values and data sources to inform the analysis based on consultation with stakeholders in Zambia.

We used the list of foods consumed in the 2015 LCMS and the Zambia CPI for the CotD analysis. We included the input parameters noted in the Supplemental Methods in the CotD software to determine the lowest cost diet that met micronutrient needs without and with fortified foods. We modeled vitamin A in sugar and edible oil; bread and maize flour fortified with vitamin A, thiamin, riboflavin, niacin, vitamin B-6, folic acid, vitamin B-12, iron, and zinc; and rice fortified with all micronutrients listed except riboflavin. The rationale for modeling fortified foods in the CotD software is that the diet cost may be less with fortified foods than with unfortified foods because fewer costly foods would need to be included in the diet to meet micronutrient needs. The fortified foods would theoretically make a greater contribution toward meeting nutrient requirements. We detail the constraints considered for modeling the cost and affordability of the nutritious diet with fortifiable foods in the Supplemental Methods.

All fortified foods were modeled at a price 2% higher than the unfortified versions, to take the industry costs for fortification into account, considering the maximum cost increase that may be acceptable to industry [6]. We determined the average daily household expenditure for food items, including equivalent monetary values for own production, gifts, and food for work, from the 2015 LCMS. Expenditure was adjusted by the CPI food price index to February 2023 prices and to the 95th percentile. We used the results of the cost of a micronutrient adequate diet and the information on household expenditure to determine the percentage of the population who would not be able to afford the micronutrient adequate diet without and with fortified foods. This is often referred to as the affordability gap.

### Feasibility of and improvements to the methodology

We used qualitative methods to assess the feasibility of and necessary improvements to the methodology. We collected data through virtual individual and group meetings and written comments, provided by country collaborators and country stakeholder groups, on the LSFF guides that describe the methodology, and via Google spreadsheets and Google forms. We purposively selected key stakeholders in Nigeria and Zambia to participate in the respective country LSFF stakeholder groups based on their experience with and/or knowledge of *1*) micronutrient deficiencies, micronutrient intake, and/or government programs to address micronutrient malnutrition; *2*) LSFF programs in the respective countries; *3*) the types and availability of existing data needed for the secondary analysis for the implementation of the methodology; and *4*) markets, particularly national and subnational markets related to fortifiable foods.

We invited representatives of government regulatory agencies, food fortification partners, United Nations organizations supporting LSFF, fortified food manufacturers, premix producers/suppliers, and academia, to participate in the stakeholder groups (for additional details on the types of individuals invited to participate, see the Supplemental Methods). We obtained stakeholders’ verbal, informed consent for meeting participation. We held a series of meetings with each stakeholder group to introduce the activity, share preliminary findings, obtain feedback and inputs on the modeling parameters, share results, and hold “pause-and-reflect” sessions to obtain feedback and inputs on the piloting process and stakeholder group meetings using real-time collaborative web platforms, including Google Jamboard and Padlet (Wallwisher).

In addition to the meetings, we sent a brief survey to the stakeholder groups to obtain feedback on modeling parameters, conducted 1-on-1 sessions with members to discuss the modeling parameters, and shared the draft Operational Overview and the Methods Guide with members for their comments and feedback. We analyzed the results qualitatively, based on themes that emerged from the data. One team member trained in qualitative data analysis identified the themes and conducted the data-driven coding, analysis, and synthesis in Excel, followed by team review and feedback.

Every study team member documented their levels of effort (hours spent) for each stage of the assessment. We used the level of effort and budget information to estimate the cost to implement the methodology. The estimated costs included the level of effort for technical and managerial personnel based in the United States, United Kingdom, Switzerland, South Africa, Nigeria, and Zambia for *1*) development of the protocol; *2*) data acquisition, cleaning, preparation, and analysis; *3*) virtual meetings with the local stakeholder groups; and *4*) report writing, technical review, editing, and formatting. The estimated costs did not include overhead costs, Negotiated Indirect Cost Rate Agreement and other organizational fees, international and local travel costs (stakeholder meetings took place virtually), or data collection, given the methodology uses existing data.

Overall, we brought together the findings from our various data sources using Excel spreadsheets and PowerPoint presentations. We presented and discussed the findings with stakeholders and integrated their feedback as part of the stakeholder sharing, internal review, and final report preparation process.

### Ethical review

The JSI Institutional Review Board determined the Nigeria and Zambia studies were exempt from human subjects oversight based on Code of Federal Regulations 46.101(b)(2), which covers survey activities without identifiers or sensitive questions that could result in harm; no participants in the study were younger than 18 y. The Nigeria National Health Research Ethics Committee, the Zambia Tropical Diseases Research Centre, and the Zambia National Health Research Authority approved the study.

## Results

### Needs assessment

**FIGURE 1:**
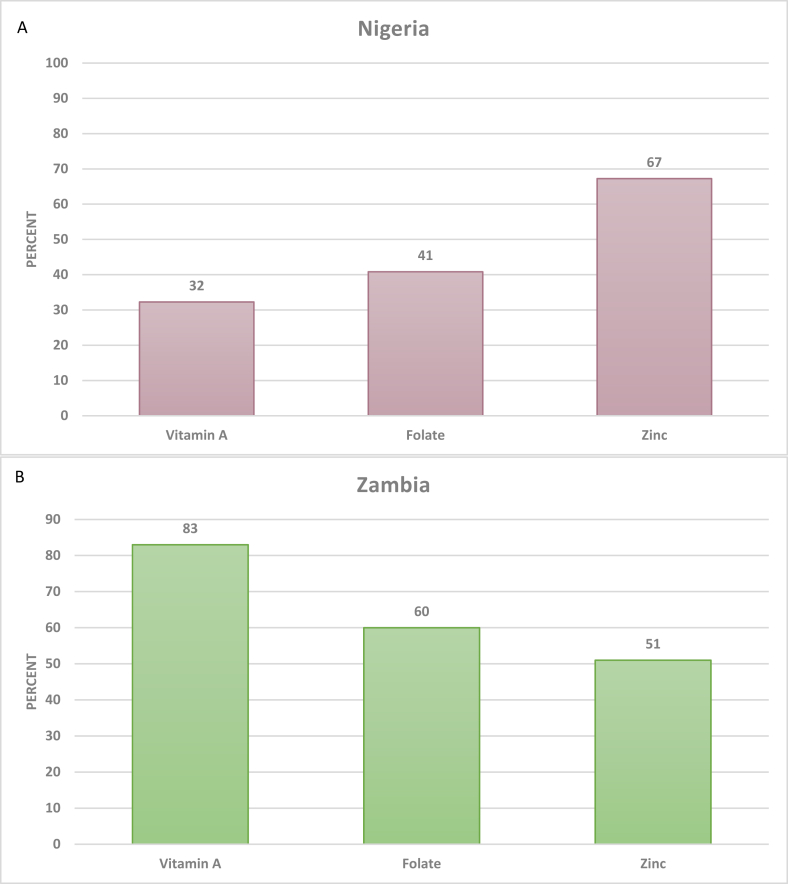
National prevalence of inadequate micronutrient intake per AFE without LSFF in Nigeria (A) and Zambia (B). Nigeria, *n* = 22,116; Zambia, *n* = 12,234. AFE, adult female equivalent; LSFF, large-scale food fortification.

Without accounting for LSFF, the national prevalence of inadequate vitamin A intake per AFE was 32% in Nigeria and 83% in Zambia (Figure 1). Folate inadequacy was 41% in Nigeria and 60% in Zambia, and the prevalence of inadequate zinc intake per AFE was 67% in Nigeria and 51% in Zambia. For most micronutrients, there was substantial variation in the prevalence of inadequacy by quintiles of household SES in urban and rural areas. In Nigeria, the largest disparity in inadequacy was for vitamin A, ranging from 13% among rural households in the highest SES quintile to 74% among rural households in the lowest SES quintile (Supplemental Figure 1). In Zambia, inadequate zinc intake per AFE ranged from 23% among urban households in the highest SES quintile to 72% among urban households in the lowest SES quintile (Supplemental Figure 2).

### LSFF design/redesign: consumption of fortifiable foods

Table 4 shows the percentage of households reporting any consumption (or acquisition, for Zambia) of the potentially fortifiable (i.e. purchased) food vehicles, including processed food products containing the food vehicle, during the recall period as well as median consumption quantities among consumers. In Nigeria, at the national-level, 74% of households reported consuming refined oil, 74% reported consuming sugar, 77% consumed wheat flour and its products, 16% maize flour, and 80% rice. Among rural households, apparent consumption of refined oil, wheat flour, and rice was more commonly reported among high SES than that among low SES households, whereas apparent consumption of sugar and maize flour were more similar across SES. Among urban households and except for maize flour, over 70% of households in every SES quintile reported apparently consuming each of these potentially fortifiable food vehicles. Quantities of refined oil and wheat flour apparently consumed were generally higher among urban households in the higher quintiles of SES than those among other population groups. There was not much variation across SES in urban and rural areas in sugar consumption, although median apparent quantities of maize flour consumed per AFE were the highest among the rural poor and apparent rice consumption was the lowest among urban and rural households in the lowest quintile of SES.

**TABLE 4:** Reach and apparent consumption of potentially fortifiable foods.

	Households consuming^1^ (%)					Median apparent consumption among consumers (g/d) per AFE				
	Refined oil	Sugar	Wheat flour	Maize flour	Rice	Refined oil	Sugar	Wheat flour	Maize flour	Rice
Nigeria										
National (*n* = 22,116)	74	74	77	16	80	7	6	32	66	73
Rural lowest SES (*n* = 3061)	56	66	36	8	49	4	6	14	75	53
Rural lower middle SES (*n* = 3060)	68	75	59	9	64	5	6	19	76	70
Rural middle SES (*n* = 3061)	72	76	73	9	72	6	7	24	75	75
Rural upper middle SES (*n* = 3060)	74	74	80	11	80	7	6	29	75	78
Rural highest SES (*n* = 3057)	73	69	87	13	85	9	6	41	68	78
Urban lowest SES (*n* = 1362)	73	79	71	25	86	5	5	21	68	66
Urban lower middle SES (n = 1362)	81	79	88	26	94	7	5	29	49	73
Urban middle SES (*n* = 1362)	83	78	92	26	96	8	6	37	45	71
Urban upper middle SES (*n* = 1362)	84	79	93	27	96	10	6	45	49	71
Urban highest SES (*n* = 1362)	80	72	92	21	87	12	7	54	62	73
Zambia										
National (*n* = 12,234)	86	72	60	49	21	18	22	26	203	41
Rural lowest SES (*n* = 1302)	46	18	8	11	1	5	10	4	128	7
Rural lower middle SES (*n* = 1308)	79	47	24	21	4	7	12	6	203	10
Rural middle SES (*n* = 1307)	92	70	44	26	7	11	15	8	191	21
Rural upper middle SES (*n* = 1308)	95	86	62	29	14	18	19	12	217	18
Rural highest SES (*n* = 1307)	96	94	79	46	34	27	26	25	195	44
Urban lowest SES (n = 1141)	88	75	61	65	15	15	19	12	211	12
Urban lower middle SES (*n* = 1140)	95	92	86	81	30	26	27	26	216	18
Urban middle SES (*n* = 1141)	94	92	92	85	40	27	27	46	195	42
Urban upper middle SES (*n* = 1140)	96	90	94	88	43	29	30	56	204	53
Urban highest SES (*n* = 1140)	93	87	94	85	55	28	36	65	194	74

Abbreviations: AFE, adult female equivalent; SES, socioeconomic strata.

1 In Nigeria, households reporting any consumption of the food vehicle (or processed food containing the food vehicle), from household purchases, during the recall period were categorized as consuming the food vehicle. In Zambia, households reporting acquiring any quantity of the food vehicle (or processed food containing the food vehicle), from household purchases, during the recall period were categorized as consuming the food vehicle.

In Zambia, 86% of households reported apparently consuming refined oil, 72% apparently consumed sugar, 60% reported consumption of wheat flour and wheat flour products, 49% maize flour, and 21% rice. Less than half of rural households in the lowest quintile of SES reported apparent refined oil consumption, whereas apparent refined oil consumption ranged from 79% to 96% among all other population strata. Similarly, apparent sugar consumption ranged between 18% and 47% among rural households in the lowest 2 SES quintiles, whereas it ranged from 70% to 94% among all other population groups. Across quintiles of SES, apparent consumption of wheat flour, maize flour, and rice were more commonly reported among urban than rural households. Among rural consumers, median apparent quantities of refined oil, sugar, wheat flour, and rice were generally largest among rural households in the highest quintile of SES and among urban consumers in the 2 highest quintiles of SES. There were no patterns in maize flour consumption across quintiles of SES in urban and rural areas.

### LSFF design/redesign: the contribution of LSFF

#### Vitamin A

**FIGURE 2:**
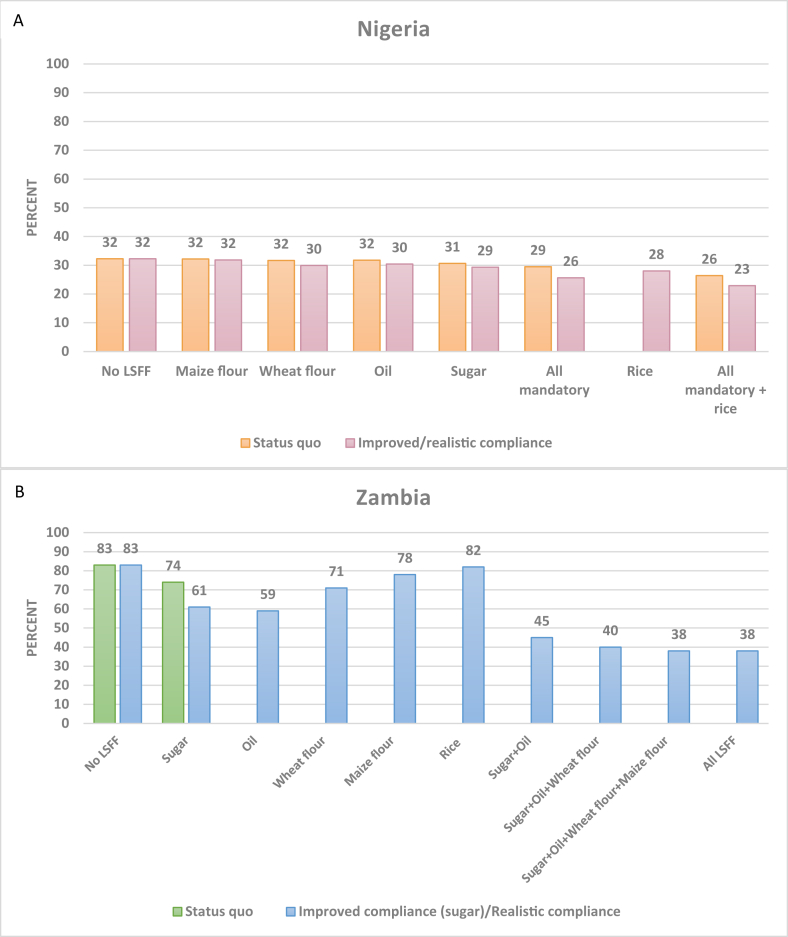
National prevalence of inadequate vitamin A intake per AFE without and with LSFF in Nigeria (A; *n* = 22,116) and Zambia (B; *n* = 12,234). AFE, adult female equivalent; LSFF, large-scale food fortification.

The prevalence of inadequate vitamin A intake per AFE without and with the contribution of LSFF are shown in Figure 2. In Nigeria (Figure 2A) under both the status quo and improved compliance scenarios, each food vehicle mandatorily fortified with vitamin A (oil, sugar, wheat flour, and maize flour) was predicted to individually bring about a small reduction in the prevalence of inadequacy (0–3 percentage points). The combination of all mandatory vitamin A fortification was predicted to reduce the prevalence of inadequacy by 3 percentage points under the status quo scenario (to 29%) and by 6 percentage points (to 26%) if compliance improved. If Nigeria additionally mandated the fortification of rice with vitamin A and could achieve a compliance rate in which 80% of fortifiable rice (assuming 30% of rice in the food system is fortifiable) was fortified to the standard, inadequacy was predicted to drop to between 23% and 26%, depending on assumed compliance of currently mandatorily fortified food vehicles. Note that across all food vehicles and combinations of food vehicles, there was no predicted risk of vitamin A intake, in the form of preformed retinol, exceeding the H-UL.

In Zambia (Figure 2B), mandatory fortification of sugar with vitamin A was predicted to reduce the prevalence of inadequate vitamin A intake per AFE from 83 to 74% under the status quo scenario and to 61% if compliance improved. If Zambia additionally mandated the fortification of oil, wheat flour, maize flour, and rice with vitamin A and could achieve compliance in which 80% of these fortifiable foods were fortified to the hypothetical national standard, inadequacy was predicted to decline to 38%. Individually, maize flour and rice fortification were each predicted to have a very small impact on vitamin A inadequacy, given low consumption of industrially milled maize flour and rice. Sugar and oil fortification alone were predicted to reduce inadequacy to 45%, and adding wheat flour was predicted to further reduce inadequacy to 40%. Across all food vehicles and combinations of food vehicles, predicted risk of vitamin A intakes above the H-UL was <5% (data not shown).

Subnationally, among households in lower SES quintiles in both urban and rural areas in Nigeria, existing LSFF was predicted to reduce vitamin A inadequacy, but even if vitamin A–fortified rice were introduced in Nigeria, the prevalence of inadequacy among these more vulnerable households was predicted to remain high (Supplemental Figure 3A). In Zambia, sugar fortification was predicted to make minor contributions to reducing vitamin A inadequacy among households in the 2 lowest SES quintiles in rural areas, but the prevalence of vitamin A inadequacy among these more vulnerable households would remain >85% (Supplemental Figure 3B).

#### Folate

**FIGURE 3:**
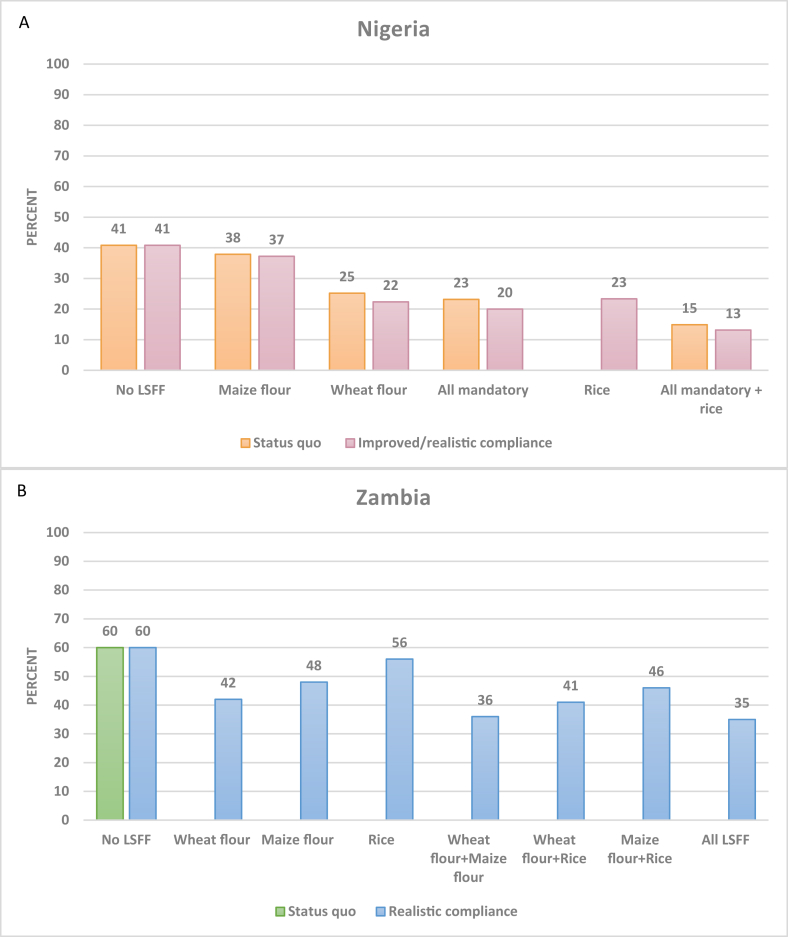
National prevalence of inadequate folate intake per AFE without and with LSFF in Nigeria (A; *n* = 22,116) and Zambia (B; *n* = 12,234). AFE, adult female equivalent; LSFF, large-scale food fortification.

In Nigeria, predicted folate inadequacy was approximately halved by the combination of wheat flour and maize flour fortification and could further decline to between 13% and 15% with the addition of folic acid-fortified rice (Figure 3A). There was no predicted risk of folic acid intake above the H-UL in any of the modeled scenarios in Nigeria (data not shown).

Folic acid–fortified wheat flour was predicted to reduce inadequate intake from 60% to 42% in Zambia (Figure 3B). The potential contributions of various combinations of folic acid-fortified wheat flour with other food vehicles (rice and maize flour) were predicted to further decrease the prevalence of folate inadequacy, and the combination of wheat flour, maize flour, and rice fortification is predicted to reduce inadequate folate intake per AFE to 35%. The predicted risk of folic acid intakes above the H-UL did not exceed 3% for any combination of food vehicle fortification (data not shown).

As previously noted, there were large differences in the prevalence of inadequacy in folate intake by household SES in Nigeria without LSFF, and this pronounced degree of inadequacy remained across SES, especially among rural households, with existing mandatory fortification (Supplemental Figure 4A). However, adding folic acid-fortified rice was predicted to reduce the prevalence of folate inadequacy to ∼10%–15% or lower among all households except for rural and urban households in the lowest SES. Across SES quintiles in Zambia, the largest predicted impacts of folic acid fortification were among urban households and among the households in the highest SES quintile in rural areas (Supplemental Figure 4B).

#### Zinc

**FIGURE 4:**
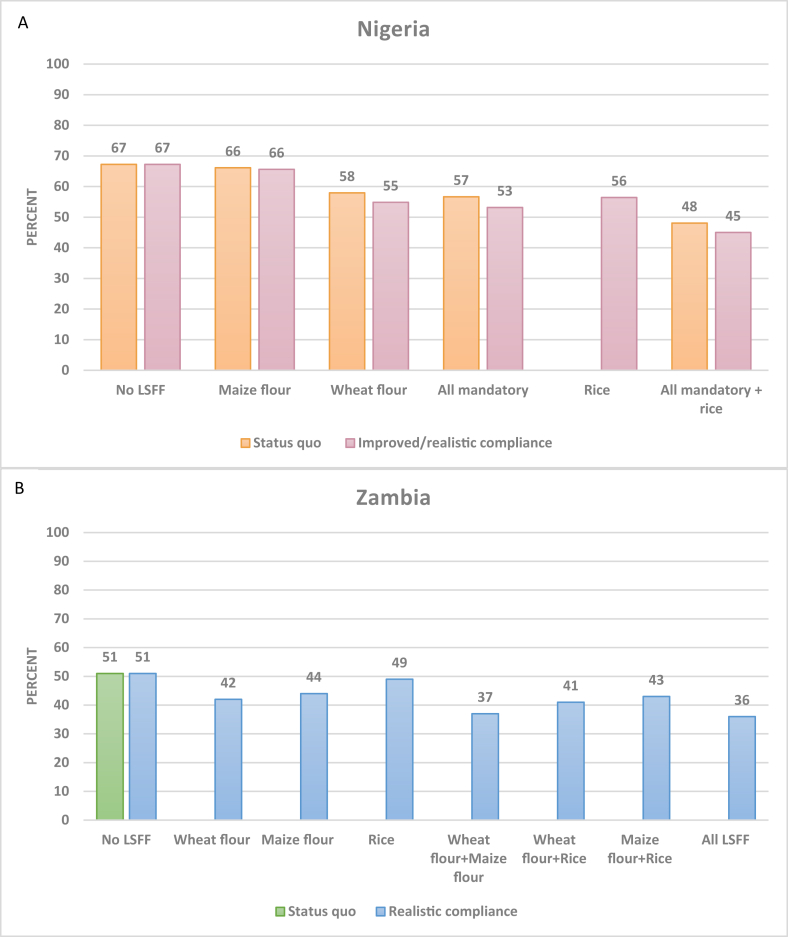
National prevalence of inadequate zinc intake per AFE without and with LSFF in Nigeria (A; *n* = 22,116) and in Zambia (B; *n* = 12,234). AFE, adult female equivalent; LSFF, large-scale food fortification.

Inadequate zinc intake per AFE in Nigeria was predicted to decline from 67% to between 53% and 57% with maize and wheat flour fortification based on the status quo and improved compliance scenarios, and the addition of zinc-fortified rice was predicted to reduce zinc inadequacy to ∼45% (Figure 4A). In Zambia, zinc-fortified wheat flour was predicted to reduce the national prevalence of zinc inadequacy from 51 to 42%, maize flour to 44%, and rice to 49% (Figure 4B). Together, fortified wheat flour, maize flour, and rice were predicted to reduce zinc inadequacy to 36%.

In Nigeria, the predicted reduction in zinc inadequacy as a result of existing wheat and maize flour fortification was, in general, larger among urban than rural households, whereas the addition of rice fortification could reduce inadequacy by an additional 5–11 percentage points across population groups (Supplemental Figure 5A). The predicted reduction in inadequate zinc intakes by quintiles of SES in Zambia was much larger among urban households compared to rural households (Supplemental Figure 5B).

### LSFF design/redesign: market availability of fortifiable foods

In our Nigerian market analysis, at the national-level and across geopolitical zones and vendor/retail outlet types, we found that all wheat flour and most of the edible oil and sugar product volume (among processors and brands identified through the existing data) was from large, local producers (60%–100%) and thus assumed to be fortifiable (TABLE 5, TABLE 6). An exception was edible oil in the North East Zone, where less than half of product volume was from large, local producers and thus considered fortifiable. Most of the maize flour product volume was locally milled by producers of small, medium, or unknown size and not considered fortifiable, except for the North Central and South South zones, where 85%–100% of the product volume was from large, local producers. Locally milled maize flour from other producers was predominant among most vendor types, except kiosks and market stalls, where the majority was from large, local producers.

**TABLE 5:** Percentage of food vehicle product volume that is fortifiable, nationally and by geopolitical zone—Nigeria.

Food vehicle	Percent fortifiable volume (large producer volume)						
	National	North Central	North East	North West	South East	South South	South West
Oil	80	79	46	66	89	94	90
Sugar	84	92	87	85	60	86	82
Wheat flour	100	100	100	100	100	100	100
Maize flour	7	100	2	33	6	85	5

**TABLE 6:** Percentage of food vehicle product volume that is fortifiable, by vendor type—Nigeria.

Food vehicle	Percent fortifiable volume (large producer volume)					
	Bakery^1^	Grocery/retail^2^	Kiosk^3^	Market stall^4^	Supermarket^5^	Wholesaler^6^
Oil	66	97	78	77	76	70
Sugar	80	96	93	95	88	73
Wheat flour	100	100	100	100	100	100
Maize flour	1	3	100	97	29	4

1 Bakery is an establishment that produces and sells flour-based baked goods such as bread, cookies, cakes, doughnuts, bagels, pastries, and pies, which are made in an oven.

2 Grocery/retail shop is a formal business but smaller than a supermarket and primarily focuses on selling food and beverages.

3 Kiosk is a small stable structure with over-the-counter sales of merchandize that can include food items.

4 Market stall or stand is usually a nonfixed structure that is generally open on all or several sides. Market stalls sell merchandize that can include food items.

5 Supermarket is a large self-serve formal business establishment with a wide selection of food and beverages that may also sell nonfood items.

6 Wholesaler is a person or company that sells goods in large quantities at low prices, typically to retailers.

In Zambia, local maize flour production was very high, followed by refined sugar and wheat flour, with relatively low local production of edible oil and rice (Table 7) [[49], [50]]. Maize flour, refined sugar, and wheat flour were not imported, but over half of the edible oil and rice supply in Zambia was imported (Table 8). In Zambia, we identified 9 edible oil producers, 5 of which were large and assumed to be capable of producing fortified oil. Among 4 sugar producers, 1 was large and considered able to fortify, whereas 3 of the 14 wheat flour millers and 17 of the 25 maize flour millers were large and considered capable of LSFF (Table 9) [51]. Most of the volume share for each food vehicle was among large producers (fortifiable, 73%–94%), except for wheat flour, where the volume share among large producers was about half. The market share of the large maize producers over the total maize flour availability in the country was estimated to be ∼70% of the domestic supply, assuming large producers use 60% of their production capacity.

**TABLE 7:** Domestic supply of foods in Zambia: local production, imports, and exports (per thousands of MT/y).

Food vehicle	Local production	Imports	Exports
Edible oil^1^ [50]	63	100	7
Sugar (refined) [49]	490	0	200
Wheat flour [49]	304	0	0
Maize flour [49]	1761	0	0
Rice [50]	35	44	7

Abbreviation: MT, metric tons.

1 For edible oil, values refer to the total supply volumes for human consumption. For imports, this refers to both unrefined edible oil for refining in Zambia and refined edible oil.

**TABLE 8:** Domestically produced and imported oil supply by oil type in Zambia^1^.

Food vehicle	Domestic production (as % of total national supply)	Imported (as % of total national supply)
Edible oil (total)	38	62
Soybean oil	26	25
Palm oil	0	36
Sunflower seed oil	9	1
Cottonseed oil	3	0

1 Values refer to the total supply volumes for human consumption. For imports, this refers to both unrefined edible oil for refining in Zambia and refined edible oil.

**TABLE 9:** Percentage of local food vehicle product volume, nationally, by producer size (large considered fortifiable) in Zambia^1^.

Food vehicle	Local producers—percentage of local production volume	
	Large producer^2^ (fortifiable), % (*n*)	Smaller producer^2^ (not considered fortifiable), % (*n*)
Edible oil	94 (5)	6 (4)
Sugar	73 (1)	27 (3)
Wheat flour	53 (3)	47 (11)
Maize flour	93 (17)	7 (8)

Abbreviation: MT, metric tons.

1 Among producers identified via existing data.

2 Edible oil: large producer defined as ≥15,000 MT/y, smaller producer defined as <15,000 MT/y; sugar: large producer defined as ≥75,000 MT/y, smaller producer defined as <75,000 MT/y; wheat and maize flour: large producer defined as ≥45,000 MT/y, smaller producer defined as <45,000 MT/y [51].

### Cost of an adequate diet

**FIGURE 5:**
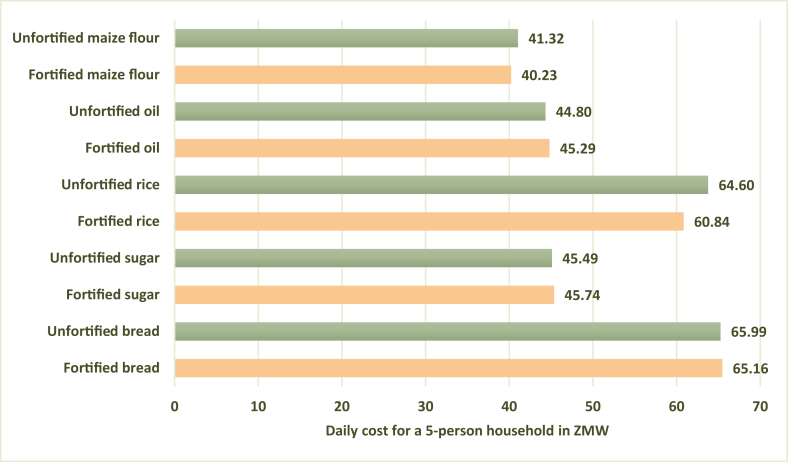
Cost of a nutritious diet without and with fortified foods modeled individually (national-level seasonal average). ZMW, Zambian kwacha.

The modeled Zambian diets with fortified maize flour, fortified rice, and fortified bread, modeled individually, showed a reduction in the national-level seasonal average daily nutritious diet cost when compared with the cost of a nutritious diet without LSFF (Figure 5). However, the cost of a nutritious diet with fortified rice and with fortified bread was a third higher than the diet containing fortified maize flour because the cost of rice and bread ranged from 4 to 8 times more than maize flour across all provinces, based on the available price data. Sugar and oil were higher cost commodities, and their inclusion in the diet slightly increased the cost in the fortified versions.

When fortified maize flour replaced unfortified maize flour in the nutritious diet, the seasonal average daily cost of a nutritious diet slightly decreased across all provinces and the affordability gap (i.e. the percentage of the population who would not be able to afford the micronutrient adequate diet) generally decreased by 1–2 percentage points (Table 10). When we modeled a nutritious diet that contained fortified sugar, oil, bread, and maize flour together, by season, in the nonlean season, the inclusion of the fortified foods increased the cost of the nutritious diet by 1–2 kwacha per day in 5 provinces (Central, Lusaka, Muchinga, Southern, and Western); and decreased the cost by 1 kwacha/d in 3 provinces (Copperbelt, Luapula, and North Western). The price remained the same in 1 province (Eastern). In the Northern Province, the daily cost increased by 3 kwacha/d. The percentage of the population that could not afford a nutritious diet went up by 1–2 percentage points in 4 provinces (Lusaka, Muchinga, Northern, and Southern); went down by 1 percentage point in 2 provinces (Copperbelt and Luapula); and remained the same in 4 provinces (Central, Eastern, North Western, and Western).

**TABLE 10:** Cost and affordability of an adequate diet in Zambia, nationally and by province, without and with large-scale food fortification [LSFF; daily cost for a 5-person household in Zambian kwacha (ZMW)].

	Cost (ZMW) (seasonal average)		Affordability gap % (seasonal average)		Cost (ZMW) (nonlean season)		Affordability gap % (nonlean season)		Cost (ZMW) (lean season)		Affordability gap % (lean season)	
	No LSFF	Fortified maize flour	No LSFF	Fortified maize flour	No LSFF	Fortified oil, sugar, bread, and maize flour	No LSFF	Fortified oil, sugar, bread, and maize flour	No LSFF	Fortified oil, sugar, bread, and maize flour	No LSFF	Fortified oil, sugar, bread, and maize flour
National	41.32	40.23	42	41	56.00	57.00	55	56	59.00	58.00	58	57
Central	36.79	35.36	33	32	52.00	53.00	51	51	55.00	55.00	53	53
Copperbelt	36.12	34.98	20	19	52.00	51.00	37	36	57.00	55.00	42	40
Eastern	50.29	48.93	55	54	51.00	51.00	56	56	76.00	74.00	71	70
Luapula	29.18	27.64	40	38	45.00	44.00	58	57	50.00	50.00	61	61
Lusaka	54.29	53.63	33	33	68.00	69.00	44	45	63.00	62.00	39	39
Muchinga	41.03	39.68	51	49	63.00	65.00	70	71	61.00	60.00	69	68
Northern	30.45	29.16	43	42	47.00	50.00	63	65	47.00	45.00	63	61
North Western	33.67	32.45	32	31	48.00	47.00	47	47	56.00	55.00	53	53
Southern	35.99	35.35	36	35	59.00	61.00	57	59	48.00	47.00	49	48
Western	54.80	54.01	69	69	71.00	72.00	77	77	66.00	67.00	74	74

The fortified foods were modeled at a price that is 2% higher than the unfortified versions, to consider the cost of fortification.

The lean season modeled diet with the same fortified foods decreased the cost of a nutritious diet by 1–2 kwacha/d in 7 provinces (Copperbelt, Eastern, Lusaka, Muchinga, Northern, North Western, and Southern); and increased the cost by 1 kwacha/d in 1 province (Western). The cost stayed the same in 2 provinces (Central and Luapula). The affordability gap decreased by 1–2 percentage points in 5 provinces (Copperbelt, Eastern, Muchinga, Northern, and Southern) and remained the same in the remaining 5 provinces (Central, Luapula, Lusaka, North Western, and Western).

### Usefulness, feasibility, and improvements to the methodology

The Nigeria and Zambia stakeholder groups expressed their interest in using the guide to design/redesign fortification programs. Although stakeholders thought that each component of the methodology had value, they felt that the guidance on using HCES and food balance sheet (FBS) data and modeling to identify the fortified food combinations to decrease micronutrient inadequacy were particularly valuable to inform decision making, provide the rationale for LSFF, justify actions, and engage and incentivize the private sector to provide nutritious foods.

Stakeholders had varied opinions about the best way to present results so that they could be easily interpreted and to provide adequate information for decision making. They particularly appreciated the visualizations showing micronutrient intake and the potential impact of LSFF, including presentation by socioeconomic group in rural and urban areas and by geographic location, and clearly showing the result with and without LSFF. The Nigeria stakeholders found the results on the potential contribution of rice fortification compelling and timely, given ongoing rice fortification discussions, whereas for the Zambia stakeholders, it was the potential for oil fortification and the CotD results that were particularly impactful.

Stakeholders in both countries indicated a willingness to invest time and resources into the assessments given the evidence generated to inform policy and programming. However, Zambian stakeholders indicated that time and cost would influence their investment in the assessments, as well as whether the findings would be useful, practical, and implementable and could help address uncertainties regarding LSFF among policymakers and civil society. Ensuring staff have the technical expertise to conduct the analyses was also a consideration. Participants suggested that the methodology could be improved by including an explanation of how to select and clean the various data sources, streamlining the methods to conduct the analysis and reinforcing the cost and benefits to the producer, which USAID will consider for future iterations of the methodology.

We estimated that the cost to implement the methodology was ∼150,000 USD in Nigeria and ∼250,000 USD in Zambia, including approximately: *1*) 100,000 USD for the needs assessment, estimation of fortifiable food consumption, and modeling the contribution of LSFF to micronutrient adequacy; *2*) 50,000 USD for the market assessment; and *3*) 100,000 for the cost and affordability of an adequate diet. The time to implement the methodology was ∼6 mo from protocol approval to the report.

## Discussion

USAID Advancing Nutrition developed the Operational Overview [12] and Methods Guide [11] to provide a comprehensive overview of and step-by-step guidelines for identifying and using available secondary data to conduct a set of analyses and generate evidence to inform the design or redesign of LSFF programs. In this article, we presented selected results from piloting that methodology in Nigeria and Zambia. The needs assessment showed that, without accounting for the contributions of LSFF, over 50% of household diets could not adequately meet vitamin A and folate requirements in Zambia and zinc requirements in both countries. Although the national prevalence of vitamin A and folate inadequacy in Nigeria were lower (32% and 41%, respectively), there was substantial subnational variation in the prevalence of inadequacy, with rates of inadequacy generally higher among poor, rural populations. The results of the needs assessment clearly demonstrated gaps in micronutrient intakes in both countries and the need for LSFF, or other interventions, to fill those gaps. As such, the needs assessment was an essential component of the overall methodology.

When we consider the potential contributions of LSFF to meeting micronutrient requirements, the current situations in Nigeria and Zambia are quite different, as Nigeria mandates the fortification of multiple food vehicles with multiple micronutrients, while among the food vehicles we modeled, Zambia currently mandates only the fortification of sugar with vitamin A [[29], [30], [31], [32]]. Our modeling results showed that in Nigeria, existing LSFF at status quo levels of compliance does improve the adequacy of diets, and if compliance with the national standards could be improved, the contribution of existing LSFF could be larger. However, the difference between modeled contributions at status quo and improved compliance levels in general were modest, and the marginal contribution of maize flour to micronutrient adequacy was either zero or very small, given low coverage and consumption of fortifiable maize flour. The modeling results also suggested that for most micronutrients, the addition of rice fortification to the current set of fortified foods could further reduce inadequacy without risking high intakes. However, the feasibility, cost, and affordability of rice fortification would need to be carefully considered. Finally, the modeling showed that the contribution of existing LSFF and the potential contribution of rice fortification was not always similar across SES, and other micronutrient interventions, such as biofortification and, when feasible, supplementation may be needed to adequately reach some population groups.

In Zambia, the modeling results showed the mandatory fortification of sugar reduced vitamin A inadequacy from 83% to 74% at the status quo level of fortification with the potential to reduce inadequacy to 61% if compliance improved. We estimated that the addition of mandatory vitamin A–fortified oil to Zambia’s LSFF programming could reduce the national prevalence of inadequate vitamin A intake to 59%, and fortifying the combination of sugar, oil, and wheat flour with vitamin A could potentially reduce inadequacy to 40%. Our modeling results also showed that fortified wheat flour and maize flour could reduce folate inadequacy (from 60% to 36%) and zinc inadequacy (from 51% to 37%). Across all micronutrients, we found that the predicted contributions of LSFF to meeting micronutrient requirements were disproportionately beneficial to wealthier, urban households. The identification of alternative fortifiable food vehicles regularly consumed by rural populations and populations of lower SES could help address this issue. Additional interventions targeted to rural, low-income households, such as biofortification, supplementation, or provision of fortified staples through social protection programs, may also be necessary to supplement ongoing or potential LSFF programming to help meet still unmet needs and to promote greater equity in micronutrient adequacy [8,[33], [34]].

In both countries, these modeling results helped the stakeholder groups identify food vehicles with good potential for improving micronutrient intakes as well as food vehicles that would have limited impact among vulnerable populations. For new food vehicles with good potential for impact, we view this as a first step in setting national fortification standards; additional modeling would be needed to set specific fortification levels [6]. As previously noted, stakeholder opinions varied with respect to the best or most effective ways to present these modeling results. Our takeaways from this stakeholder feedback were that *1*) it is important to seek and incorporate stakeholder feedback on how modeling results are presented and *2*) it can be important to present modeling results in multiple formats to ensure that the results resonate across different types of stakeholders. Supplemental Figures 6–8 show examples of alternative ways that we presented results on the contribution of LSFF to micronutrient intake and adequacy during stakeholder meetings.

One of the limitations of using HCES data to model the impacts of LSFF is that strategies for identifying consumption of industrially processed, and hence fortifiable, food is limited. HCES data often distinguish between foods that are purchases and foods that are acquired in other ways (e.g. from own production or from gifts), but they do not gather information on brands and do not distinguish between the source of those purchased foods (e.g. from an industrial processing facility compared with a local, small-scale processor). Integrating analyses of market survey data can help address this shortcoming by providing information on the availability of fortifiable and fortified foods in different regions and by vendor types across the country. In Nigeria, the market survey data showed fairly widespread availability of fortifiable foods across geopolitical zones and vendor types, especially for edible oil, sugar, and wheat flour. Fortifiable maize flour availability appeared more favorable in North Central and South South zones, as well as in kiosks and market stalls, but amounts of unbranded maize flour, sold loose in these areas, may not have been captured adequately, possibly resulting in overestimation of the volume of packaged products. Low market availability of fortifiable maize flour in some zones and vendor types may limit the potential contribution of maize flour fortification to micronutrient adequacy in these areas.

The market assessment in Zambia showed that nationally, fortifiable food availability was excellent for sugar, which is already mandatorily fortified in Zambia and continues to be centrally, industrially produced. Edible oil and wheat flour appear to have potential for large-scale fortification, based on local, central, and industrial production and may be promising options. Over half of Zambia’s edible oil is imported, the majority assumed to be crude edible oil that is processed in Zambia [35]. For the remaining refined edible oil imports, fortification policies and regulations would need to include mandatory fortification of imported, refined oil. Local maize production is very high in Zambia, and maize flour appears to be a key staple food. However, the benefit of fortifying maize flour may be limited to the urban settings and higher SES households in rural areas where coverage and consumption of purchased maize flour is relatively high [36]. Rice consumption and domestic supply is low compared with the other fortifiable foods, and thus it appears that it is not currently a suitable fortification vehicle in Zambia.

The market assessments in Nigeria and Zambia were slightly different. In Zambia, we did not have existing data from a national or subnational survey on the availability of fortifiable food vehicles by province or vendor type. We relied on general information that we found through the broad agrifood information system. Despite the limited data, the results were useful to inform the broad market availability of fortifiable foods, but information from a more detailed market survey would have been helpful to better understand the situation in provinces and by types of vendors. Periodic market surveys or existing information systems that include collection of data on producer/product supply of fortifiable foods by region and vendor types can be linked to consumption data (and data on micronutrient content of fortifiable foods, if collected) to improve modeling inputs and estimates of micronutrient intake. The study demonstrated how a market assessment can provide results that practitioners can use to model the contribution of LSFF considering subnational differences in fortifiable food availability and the importance of crosschecking market findings with data from other sources and local stakeholders.

It is important to note that, due to time constraints, the market assessments and modeling were conducted concurrently, so we were unable to apply the estimates of the percentage of the food vehicle that is fortifiable from our market assessment in our modeling of the contribution of LSFF to micronutrient adequacy. For the modeling, we used estimates of the percentage of the food vehicle that is fortifiable from stakeholder group estimates, the Global Fortification Data Exchange, and industry assessments, as described in TABLE 1, TABLE 2 and in the Supplemental Materials. Supplemental Table 4 shows that most of the Nigeria market assessment estimates of the percentage of the food vehicle that is fortifiable were similar to those from the sources we used, with a few exceptions, whereas Supplemental Table 5 for Zambia shows that there were notable differences in estimates for wheat and maize flour. Additional stakeholder discussions and possibly rapid data collection could help to better understand the reasons for these differences. Given that the data sources and methods used to arrive at these estimates differ between the modeling and market assessments and we do not have a gold standard comparator, it is not possible to conclusively say one set of estimates should be preferred over the other. Given the importance of ensuring stakeholder engagement with the process and trust in the results, in the future, we recommend conducting the market assessment first, discussing the market assessment results alongside other available estimates with the stakeholder group, and then using the agreed upon values for the modeling. Sensitivity analyses could also be used to assess the impact of uncertainty in these modeling parameters on estimates on the contribution of LSFF to micronutrient adequacy. Clear and standardized market assessment indicator definitions are also needed. The differences in Zambia demonstrate the importance of using market data that has been collected to be representative at the national or subnational level to adequately inform LSFF.

Our diet cost and affordability modeling in Zambia found that although fortified maize flour would reduce the cost of a nutrient adequate diet by ∼1 kwacha/d and decrease the affordability gap across all provinces, there would be a 1–2-kwacha increase in diet cost and a slight increase in the affordability gap in about half of the provinces in the nonlean season for fortification that included wheat flour as bread, edible oil, sugar, and maize flour. However, during the lean season the CotD and unaffordability would decrease in about half of the provinces. Some possible reasons for slightly higher diet cost during the nonlean season may be due to the CotD software selecting some higher cost foods to meet calcium requirements. The CotD software models for 13 micronutrients, not all of which were considered for the LSFF scenarios, including calcium. Any nutrients that the CotD software meets at 100% of the requirement can drive up the overall diet cost. If it was feasible to model only the micronutrients included in the fortification modeling, this would enable a potentially more accurate comparison and the cost of the fortified diet may be less. Preliminary results from the 2020 Zambia National Food Consumption and Micronutrient Status Survey show a high prevalence of inadequate calcium intake among all surveyed participants [37]. It will be important for the Government of Zambia and stakeholders to explore options to improve calcium intake, including the feasibility of fortification, and include calcium in modeling scenarios.

Overall, the CotD analysis shows that there would not be dramatic changes in diet cost or affordability with LSFF, but for households with low incomes, any increase in diet cost, even slight, can affect the family and individual diets. This points to the importance of considering social protection programs to provide fortified staple foods to households vulnerable to inadequate micronutrient intake. The WFP Fill the Nutrient Gap analyses in Zambia demonstrate the potential of social protection programs to reduce the CotD [38]. Although some Zambia and Nigeria stakeholder group members felt the CotD results would be important for advocacy and policies to address LSFF food prices, given the small impact of LSFF on the cost and affordability of an adequate diet in Zambia, we suspect that the use of these results for LSFF advocacy might be limited. When modeling fortified compared with unfortified maize flour in Zambia, WFP found that the CotD went down by ∼0.8 Zambian kwacha (ZMW) per day per household [38], similar to our modeling results for fortified maize flour. WFP analyses in other countries have demonstrated a slightly larger reduction in the cost of an adequate diet when LSFF is part of general food distribution [39] or if the fortified food could be accessed on the market at the same price as the unfortified food [40]. When WFP modeled a 2% higher price for the fortified compared with unfortified food as we did in our analysis, their results were similar to ours in terms of the magnitude of change in the CotD [41]. As such, whether it would be worth spending time and resources conducting a CotD analysis in other contexts would need to be considered on a case-by-case basis and include considerations around the need for advocacy, data availability, and stakeholder feedback on its importance.

Both the piloting process and the results of the pilots should be interpreted in the context of study limitations. First, although the methodology covers the use of 24-h dietary recall data, food frequency data, and HCES data, only HCES data were used in the pilots because of their availability, so we are unable to comment on the feasibility of implementing the methodology using other data sources. In addition, given the age of the Zambia LCMS data (2015) and several study characteristics that make the data less fit to assess the micronutrient adequacy of diets (including that the survey collected data on food acquisitions rather than consumption and the data set did not include food conversion factors for nonstandard units), the accuracy of the results is uncertain. Likewise, the NLSS food consumption data lacked adequate specificity and disaggregation with respect to unrefined and refined oils, and lower-than-expected estimates of apparent consumption of several fortifiable food vehicles points to a possible need to review the survey data collection methods to ensure quantities of foods consumed are being recalled and recorded as accurately as possible. We recommend conducting these analyses with more recent data, when available, to verify the findings. For countries considering using the methodology in the future, it will be important to consider whether the quality of the data available for these analyses is adequate to answer their specific policy questions and support their decision making. It will also be important to conduct sensitivity analyses around uncertain parameters to determine their influence on the results and the corresponding implications for LSFF design. If uncertain parameters are identified during sensitivity analysis such that LSFF design decisions might change based on their value, this may warrant additional data collection to reduce uncertainty. Where possible, triangulating the findings based on dietary data with, for example, estimates of micronutrient deficiency from national micronutrient surveys, may also help lend credibility to the results.

In addition to these limitations, the results of the needs assessments and LSFF modeling in both countries are subject to the limitations inherent in using household-level data to assess the adequacy of diets, including the potential for recall error, inadequate specificity of some fortifiable foods on the food list, inadequate accounting for foods consumed away from home, and the assumption that foods are distributed within the household in proportion to age-specific and sex-specific energy requirements [[18], [42], [43]]. The way we handled some of these issues may have impacted the results. For example, the way we identified and dealt with outliers may have impacted our estimates of the risk of high micronutrient intakes. As noted in the Methods Guide, there are multiple ways to detect and correct outliers in HCES food consumption data, and future analyses should consider conducting sensitivity analysis around this analytical choice [11].

Similarly, we assessed the adequacy of the household diet relative to the micronutrient requirements of a nonpregnant, nonlactating adult female. If instead another reference group was chosen (e.g. children aged 2–5 y or adult males), the results would likely differ. In addition, neither the NLSS nor the Zambia LCMS collected information on the pregnancy status of household members, which may result in an underestimation of the prevalence of micronutrient inadequacy in households with pregnant women. As such, assessing the contribution of LSFF for meeting the micronutrient requirements of pregnant women, specifically, would require separate analyses using data better suited to modeling the micronutrient adequacy of diets among pregnant women.

In Nigeria, the data on maize flour volume used for the market assessment may not have adequately captured unbranded products, so the proportions at regional level may be skewed. This may be due to the relatively low proportion of maize from fortifiable sources (large-scale, formal, industrial producers) in several geopolitical zones, where a high proportion of unbranded products from small-scale producers may be common. The survey primarily collected data on branded products. Although it did assess packaged products vis-a-vis loose food products, thorough capturing of loose and unbranded products can be difficult. Similarly, red palm oil predominantly sold loose or directly by small-scale producers may not have been fully captured in the market assessment, resulting in an overestimation of the fortifiable proportion. In Zambia, we focused on using local production volume data, which may have underestimated the proportion of the national product volume of edible oil that is fortifiable if a large percentage of the refined oil imports are from large producers that were not considered in the categorization. We also assumed that food producer annual production capacities were proportionate to the actual annual production volume, which could overestimate the proportion of the volume that is fortifiable in the case that many small-scale producers were not identified. A limited amount of data collection among producers could validate assumptions or improve their accuracy.

Overall, we found the methodology feasible to implement within a reasonable time frame using existing data and with the support of country stakeholders, to provide practical results to inform LSFF programming. Based on this experience, we expect that the methodology would be feasible to implement in most LMIC contexts. However, in countries without nationally representative 24-h dietary recall data, semiquantitative food frequency data, or HCES data, without additional data collection, the needs assessment would need to rely on FBS data, which has limitations relative to the other secondary data sources, and it is not recommended to undertake the design step using FBS data [12]. In Nigeria and Zambia specifically, we recommend repeating the methodology, or specific aspects of the methodology, when new national dietary recall data, household food consumption data, or market data become available, to be able to inform the need for adjustments to the LSFF program. In general, we also recommend training and mentoring in the methodology for technical staff from universities, institutes, and/or statistical offices, and strategies to decrease the costs so that the methodology is more accessible to countries for their use to inform LSFF programming.

## Author contributions

The authors’ responsibilities were as follows—KPA, IW, OD, MBW: designed the research; KPA, EAG, SMJ, JLH, MJM, OMA, MBW: conducted the research; KPA, MBW: wrote the paper and had primary responsibility for final content; EAG, SMJ, JLH, MJM, OMA, JY, SA, HD, IW, OD: provided technical review of the paper; and all authors: read and approved the final manuscript.

## Data availability

Data described in the manuscript, code book, and analytic code will be made available upon request.

## Funding

The United States Agency for International Development (USAID) provided financial support for this article through its flagship multisectoral nutrition project, USAID Advancing Nutrition. It was prepared under the terms of contract 7200AA18C00070 awarded to JSI Research & Training Institute, Inc. The contents are the responsibility of JSI and do not necessarily reflect the views of USAID or the United States Government. USAID provided publication support for this article through USAID Advancing Food Fortification Opportunities to Reinforce Diets, under terms of cooperative agreement 7200AA22LE00002 awarded to TechnoServe.

## Conflict of interest

The authors report no conflicts of interest.
