# Supplementary material for: Informing the Design of Large-Scale Food Fortification Programs with Secondary Data: Pilot Results from Nigeria and Zambia
Source: Curr Dev Nutr. 2024 Dec 10;9(2):104522. doi: 10.1016/j.cdnut.2024.104522 (PMC11803876; doi:10.1016/j.cdnut.2024.104522)
Supplement: Multimedia component 1 [file mmc1.docx]

**Informing the design of large-scale food fortification programs with secondary data: Pilot results from Nigeria and Zambia**

Katherine P Adams, Emmanuel A Gyimah, Svenja M Jungjohann, Jacqueline L Hems, Musonda J Mofu, Olufolakemi Mercy Anjorin, Jennifer Yourkavitch, Silvia Alayón, Heather Danton, Ingrid Weiss, Omar Dary, and Monica B Woldt

***Supplemental Methods***

**Analyses of Household Consumption and Expenditure Survey Data**

*HCES data descriptions*

The National Bureau of Statistics (NBS) implemented the Nigeria Living Standards Survey (NLSS) with support from the World Bank. The NBS collected data from September 2018 through September 2019, and the data are representative at the national, zonal, and state levels, and by urban and rural residence (1). The Zambian Central Statistical Office implemented the Zambia Living Conditions Monitoring Survey (LCMS), with data collection between April and May of 2015 (2). The data are representative at the national level, by province, and by urban and rural residence.

*Disaggregated Nigeria refined oil consumption assumptions*

**Supplemental Table 1. Refined oil consumption assumptions**

| **Zone** | **Proportion groundnut oil (not fortifiable)^1^** | **Proportion refined palm or soybean oil (fortifiable)^1^** |
| --- | --- | --- |
| North Central | 0.4 | 0.6 |
| North East | 0.5 | 0.5 |
| North West | 0.5 | 0.5 |
| South East | 0.2 | 0.8 |
| South South | 0.2 | 0.8 |
| South West | 0.2 | 0.8 |

^1^Zone-specific assumptions based on input from Nigeria stakeholder group members collected via group discussions, individual interviews, and questionnaires.

*Assumed energy requirements and adjustments to energy requirements based on breastfeeding status*

We estimated energy requirements by age, sex, average body weight, and condition (e.g., pregnancy or lactation) for each household member based on the Human Energy Requirements recommendations from the Joint Food and Agriculture Organization of the United Nations (FAO)/World Health Organization (WHO)/United Nations University (UNU) Expert Consultation (3). For children under age 18, we assumed a moderate physical activity level. For women age 18–29.9 years and 30–59.9 years, average body weight was based on the 2018 Nigeria Demographic and Health Survey (4) and the 2013–2014 Zambia Demographic and Health Survey (5). We assumed the weight of older women and of men were in the same “category” as women for whom we had estimates. For example, if the average weight of women 18–29.9 years put them in the 3rd category of women’s weight ranges in the FAO/WHO/UNU Human Energy Requirements document, we assumed that older women were also in the 3rd category. For all adults, we assumed a physical activity level corresponding to 1.6x basal metabolic rate.

Lactation/breastfeeding status were not collected in the 2018–2019 NLSS nor the 2015 Zambia LCMS, so we made adjustments to energy requirements based on a set of assumptions. Specifically, for households with a child under 2 years of age, we randomly assigned a breastfeeding status to the child in proportion to the percentage of children under age 2 in Nigeria/Zambia who are breastfed according to the most recent Demographic and Health Survey data. For children assumed to be breastfed, we assumed there was also a lactating woman in the household. For breastfed children, we estimated their total energy requirement from complementary foods by deducting the (age-specific) energy assumed to be provided from breastmilk (6) from their daily energy requirement (note that because children under 3 months of age were assumed to be exclusively breastfed, we set their energy requirements from complementary foods to zero). For lactating women with a child under 6 months of age, we added 330 kilocalories/day to her energy requirements, while we added 400 kilocalories/day to lactating women with a child > 6 months (7). We were unable to make adjustments for energy requirements during pregnancy, as pregnancy status was not collected in either the NLSS nor LCMS survey.

**Market Assessment**

The 2021 the Global Alliance for Improved Nutrition (GAIN) and Ipsos Nigeria cross-sectional market assessment data we used included information on brand availability and market penetration of available brands of six mandatory food vehicles (we focused on sugar, oil, and wheat and maize flour in this analysis) across different vendor/retail outlet types (bakeries, grocery stores/retail shops, kiosks, market stalls, supermarkets, and wholesalers) in 24 selected states across the six geopolitical zones of Nigeria (8). To show the proportion of products from known and large producers, we analyzed the results disaggregated by origin, defined in four categories 1) imported, large producer/supplier; 2) large, local producer; 3) imported, other producer (small, medium, or unknown size); and 4) other local producer (small, medium, or unknown size). The identification of producers by size was informed by industries’ annual production data (9). We analyzed the market availability by geopolitical zone and vendor/retail outlet type.

For the Zambia market assessment, we systematically searched available data sources, including Food Balance Sheets from FAO and Zambia, industry research, agriculture research reports, producer and retailer websites and social media, online business media articles, free online portals, and previous relevant research (10-16). These data sources provided information on a) domestic supply of the food vehicles (volume) by origin (locally produced or imported) and food type (such as type of oil [palm oil, sunflower oil] or type of flour [bread or cake flour]); b) producers/suppliers and their production volume or capacity; c) branded products (such as Zambia’s Pride triple refined sunflower oil) by food type; d) food channels (such as food services, industrial use, retail, or retail outlet types), and e) regional availability. **Supplemental Table 2** shows the various data sources and the type of data provided by each source for the Zambia market assessment (16).

**Supplemental Table 2. Data sources and types of data provided for the Zambia Market Assessment**

| **Data sources** | **Disaggregation categories for volume data** | | | | |
| --- | --- | --- | --- | --- | --- |
|  | **Domestic supply** | **Producers/**  **Suppliers** | **Brand products** | **Food channels** | **Regional availability** |
| FAO Food Balance Sheets 2023; Zambia Food Balance Sheets 2018–2019 | ✓ |  |  |  |  |
| Free online portals | ✓ | ✓ |  |  |  |
| Industry research: | ✓ | ✓ |  |  |  |
| Agriculture research reports | ✓ | ✓ |  |  |  |
| Producer and retailer websites, Facebook pages, and LinkedIn posts | ✓ | ✓ | ✓ | ✓ | ✓ |
| Online business media articles | ✓ | ✓ |  |  |  |
| Previous relevant research | ✓ | ✓ |  |  |  |

Source: Global Alliance for Improved Nutrition (16)

We used criteria developed by USAID to identify producers with a sufficiently large volume to have the capacity for large-scale industrial fortification (17) **(Supplemental Table 3).**

**Supplemental Table 3. Criteria to Define a Large-Scale Producer in Low-Income Countries**

| **Food item** | **Metric tons per hour** | **Metric tons per day** | **Metric tons per year** |
| --- | --- | --- | --- |
| Sugar | 20 | 500 | 75,000 |
| Oil | 5 | 50 | 15,000 |
| Wheat flour | 20 | 150 | 45,000 |
| Maize flour | 20 | 150 | 45,000 |
| Rice | 10 | 100 | 30,000 |

Source: USAID (17)

**Estimates of the “Percentage of the Food Vehicle that is Fortifiable” from our Market Assessment and the Sources We Used for Modeling**

When modeling the contribution of large-scale food fortification (LSFF) to micronutrient adequacy, one key modeling parameter is the percentage of the food vehicle that is fortifiable. Our market assessments in Nigeria and Zambia, through the analysis of existing data, estimated the percentage of the food vehicle that is fortifiable at national and sub-national levels. We defined the percentage as the proportion of the total supply volume from large producers/suppliers (for Nigeria, local and imported, and for Zambia, only local). However, given time constraints, the market assessment and modeling were conducted concurrently. For modeling the contribution of LSFF to micronutrient adequacy, we used national estimates of the percentage of the food vehicle that is fortifiable from industry assessments (9, 15), the Global Fortification Data Exchange (GFDx) (18), and input from the stakeholder groups. We also discussed and agreed upon the estimates with the respective stakeholder group members.

**Supplemental Table 4** (Nigeria) and **Supplemental Table 5** (Zambia) compare the estimates from our market assessment with those we used in our modeling. The table footnotes provide descriptions of the indicator for each source. In Nigeria, most of our market assessment values for the percentage of the food vehicle that is fortifiable were similar to those from the other sources that we used (within 20 percentage points), except for edible oil in the North East zone, sugar in the South East zone, and maize flour in the South South and North Central zones, where differences were high, by 34 to 80 percentage points. For Zambia, the differences in the estimates of the percentage of the food vehicle that is fortifiable were most striking for wheat flour and maize flour, with differences ranging from 46 percentage points for wheat flour to 57 percentage points for maize flour.

**Supplemental Table 4. Percentage of Food Vehicle that is Fortifiable: Comparison of Estimates from “Sources Used” (Industry Assessments, GFDx, or Stakeholder Group Estimates) and the Market Assessment, Nationally and by Geopolitical Zone, Nigeria**

|  | **Edible Oil** | **Sugar** | **Wheat flour** | **Maize flour** |
| --- | --- | --- | --- | --- |
| **National** |  |  |  |  |
| Sources used^1^ | 80^2^ | 100 | 96 | 20 |
| Market assessment | 80 | 84 | 100 | 7 |
|  |  |  |  |  |
| **North Central** |  |  |  |  |
| Sources used^3^ | 80^2^ | 100 | 96 | 20 |
| Market assessment | 79 | 92 | 100 | 100 |
|  |  |  |  |  |
| **North East** |  |  |  |  |
| Sources used^3^ | 80^2^ | 100 | 96 | 20 |
| Market assessment | 46 | 87 | 100 | 2 |
|  |  |  |  |  |
| **North West** |  |  |  |  |
| Sources used^3^ | 80^2^ | 100 | 96 | 20 |
| Market assessment | 66 | 84 | 100 | 33 |
|  |  |  |  |  |
| **South East** |  |  |  |  |
| Sources used^3^ | 80^2^ | 100 | 96 | 20 |
| Market assessment | 89 | 60 | 100 | 6 |
|  |  |  |  |  |
| **South South** |  |  |  |  |
| Sources used^3^ | 80^2^ | 100 | 96 | 20 |
| Market assessment | 94 | 86 | 100 | 85 |
|  |  |  |  |  |
| **South West** |  |  |  |  |
| Sources used^3^ | 80^2^ | 100 | 96 | 20 |
| Market assessment | 90 | 82 | 100 | 5 |

^1^Sources used include: Oil (national): Ayodele Tella, TechnoServe, personal communication, Nigeria, April 20, 2023, based on data collected by TechnoServe on the volume of edible oil processed by large producers; Sugar (national): Assumption/ Stakeholder input; Wheat flour (national): Global Fortification Data Exchange (18) (source as noted in GFDx: Titilola Abolade, Nutrition International, Personal communication, Nigeria, 2021); Maize flour (national): Global Fortification Data Exchange (source as noted in GFDx: Talatu Ethan, Standards Organization of Nigeria, Personal communication, Nigeria, 2017). GFDx estimates are based on the percentage of the total food vehicle, in metric tons, processed by an industrial processor and is required to be fortified according to relevant legislation or could be fortified through industrial food processing if there is no mandatory fortification in a country.

^2^The estimate of fortifiable refined oil excludes groundnut oil, which is refined by small scale industries in Nigeria and is not feasible for fortification.

^3^Note that for “Sources used” the zone values are assumed to be the same as the national values.

**Supplemental Table 5. Percentage of Food Vehicle that is Fortifiable: Comparison of Estimates from “Sources Used” (Industry Assessments, GFDx, or Stakeholder Group Estimates) and the Market Assessment, Nationally, Zambia**

|  | **Edible Oil** | **Sugar** | **Wheat flour** | **Maize flour** |
| --- | --- | --- | --- | --- |
| Sources used^1^ | 70 | 90 | 100 | 35 |
| Market assessment^2^ | 94 | 73 | 54 | 92 |

^1^Sources used include: Edible oil and sugar: TechnoServe 2023, based on the share of local processing done by industrial/large-scale processors; Wheat flour: Zambia Stakeholder Group input; Maize flour: Global Fortification Data Exchange (18) (source as noted in GFDx: Southern African Development Community, Government of South Africa's Department of Health, Smarter Futures, UNICEF. Regional Consultative and Capacity Building Workshop on Strengthening Food Fortification Programs: Monitoring and Surveillance Systems. South Africa. 2018). GFDx estimates are based on the percentage of the total food vehicle, in metric tons, processed by an industrial processor and is required to be fortified according to relevant legislation or could be fortified through industrial food processing if there is no mandatory fortification in a country.

^2^Note that market assessment estimates were based on data from 4 sugar millers, 9 edible oil producers, 14 wheat flour millers, and 25 maize flour millers. Market assessment results are based on the proportion of the local production volume share from large producers.

**Cost of the Diet**

*Input parameters*

The cost of the diet (CotD) analysis was primarily informed by the 2015 LCMS food list. We identified foods in the list that are not usually consumed by specific household members, such as breastfed children 6–23 months of age or pregnant women, through a review of secondary data such as the 2018 Zambia Demographic and Health Survey (19) and discussions with stakeholders in Zambia. We used the nutritional constraints in the CotD software (20) for the modeled household, which include average energy requirements and recommended intakes of protein, fat, and 13 micronutrients as indicated in the 2004 report from FAO, WHO, and UNU (3) for human energy requirements and the 2004 WHO and FAO report for micronutrient intake (21). For analysis without LSFF, we used the CotD software default minimum and maximum acceptable daily food portions. We adjusted the constraints for maize to create the staple-adjusted nutritious diet, referred to as the “nutritious diet”. Maximum constraints are limited within the software so as not to exceed the percentage of energy provided by foods. We calculated the food price per 100 grams or milliliters in local currency. We used the data from the food composition table (FCT) built into the CotD software. Most food matches were taken from the West Africa, Kenya, Senegal, and U.S. Department of Agriculture FCTs in the CotD database. Where foods did not exist or an appropriate match could not be found, we created the new food in the CotD FCT from the Zambia FCT (22). We used two CPI data price points for the analysis—August 2022 to represent the non-lean season and February 2023 to represent the lean season.

*Constraints*

The following constraints were implemented to model the cost and affordability of the nutritious diet with fortifiable foods in Zambia:

- We modeled fortified maize flour, bread, and rice in separate models, with a minimum of two daily portions and a maximum of three daily portions (except for the child 6–23 months where the minimum was one daily portion).
- Rice was not included in Muchinga Province because prices were not available for both seasons.
- Edible oil was included as a minimum of one daily portion for all family members.
- Sugar was limited to one daily portion for all family members.
- Bread and maize flour were modeled with varying constraints and combinations of foods, to achieve a total minimum of two daily portions and a total maximum of three daily portions (except for the child 6–23 months where the total minimum was one daily portion).

**Feasibility of and improvements to the methodology**

*Stakeholder groups*

The following types of individuals were invited to participate in the stakeholder groups: a) government staff from the Ministry of Health; Ministry of Agriculture; Ministry of Commerce, Trade, and Industry; national standards agency; and national laboratories (e.g., micronutrient analysis of food samples and/or biological samples); b) nongovernmental organizations working in LSFF and/or micronutrient nutrition; c) academic professionals with expertise in LSFF and/or micronutrient nutrition; d) private sector, including representatives from industry groups or companies producing fortifiable foods and micronutrient premix providers; e) donors supporting LSFF; and f) United Nations organizations supporting LSFF (e.g., UNICEF, World Food Program, and the World Health Organization).

***Supplemental Figures***

**Supplemental Figure 1**. Nigeria baseline prevalence of inadequate vitamin A (panel A), folate (panel B), and zinc (panel C) intake per AFE without LSFF by quintiles of household SES in urban and rural areas (n=22,116)

**Supplemental Figure 2**. Zambia baseline prevalence of inadequate vitamin A (panel A), folate (panel B), and zinc (panel C) intake per AFE without LSFF by quintiles of household SES in urban and rural areas (n=12,234)

**Supplemental Figure 3.** Prevalence of inadequate vitamin A intake per AFE without and with LSFF by quintiles of household SES in urban and rural areas in Nigeria (panel A, n=22,116) and Zambia (panel B, n=12,234)

**Supplemental Figure 4.** Prevalence of inadequate folate intake per AFE without and with LSFF by quintiles of household SES in urban and rural areas in Nigeria (panel A, n=22,116) and Zambia (panel B, n=12,234)

**Supplemental Figure 5.** Prevalence of inadequate zinc intake per AFE without and with LSFF by quintiles of household SES in urban and rural areas in Nigeria (panel A, n=22,116) and Zambia (panel B, n=12,234)

**Supplemental Figure 6.** Vitamin A Apparent Intake Population Percentile Curves and Estimates of Dietary Inadequacy by Residence in Nigeria (n=22,116)

**Supplemental Figure 7.** Prevalence of Vitamin A Inadequacy in Nigeria without and with LSFF by Geopolitical Zone (n=22,116)

Supplemental Figure 8. Dietary Gap in Vitamin A Adequacy at the 25^th^ Percentiles of Apparent Vitamin A Intake Per AFE in Nigeria (n=22,116)

**References**

1. The World Bank. Living standards survey 2018-2019, Nigeria. 2022. Available from: <https://microdata.worldbank.org/index.php/catalog/3827>.

2. Central Statistical Office. Living conditions monitoring survey vii (LCMS) 2015. [dataset]. ZMB_2015_LCMS-VII_v01_M. 2015. Available from: <https://catalog.ihsn.org/catalog/7105>.

3. Food and Agricultural Organization of the United Nations, World Health Organization. Human energy requirements. Report of a Joint FAO/WHO/UNU Expert Consultation Rome,17–24 October 2001. Rome, 2004.

4. National Population Commission - NPC/Nigeria and ICF. Nigeria Demographic and Health Survey 2018. [dataset]. Ngpr7afl.Dta. Abuja, Nigeria, and Rockville, Maryland, USA: NPC and ICF, 2018.

5. Central Statistical Office, Ministry of Health Zambia, ICF International. Zambia Demographic and Health Survey 2013-2014. [dataset]. ZMPR61FL.DTA. Rockville, Maryland, USA: Central Statistical Office/Zambia, Ministry of Health/Zambia, and ICF International, 2014. Available from: <https://dhsprogram.com/data/dataset/Zambia_Standard-DHS_2013.cfm>.

6. Brown K, Dewey K, Allen L. Complementary feeding of young children in developing countries: A review of current scientific knowledge. World Health Organization: Geneva, 1998.

7. U.S. Department of Agriculture and U.S. Department of Health and Human Services. Dietary guidelines for americans, 2020-2025. 9th edition. 2020. Available from: <https://www.dietaryguidelines.gov/sites/default/files/2021-03/Dietary_Guidelines_for_Americans-2020-2025.pdf>.

8. Global Alliance for Improved Nutrition. Market level assessment of the fortification status of mandatory food vehicles in Nigeria. Unpublished report. 2021.

9. TechnoServe. Nigeria large-scale food fortification (LSFF) opportunity assessment. Opportunities to expand large-scale food fortification (LSFF) in Nigeria in collaboration with the food industry. PowerPoint presentation. April 2023.

10. Karuho O. Institutionalizing national food balance sheet. Case of Zambia. 2020. Available from: <https://agra.org/wp-content/uploads/2020/10/Food-Balance-Sheet-Case-of-Zambia.pdf>.

11. Food and Agriculture Organization of the United Nations. FAOSTAT. 2023. Available from: <https://www.fao.org/faostat/en/>.

12. Sutton J, Langmead G. An enterprise map of Zambia. London, UK: International Growth Centre, 2013. Available from: <https://www.theigc.org/sites/default/files/2014/04/An-Enterprise-Map-of-Zambia-English.pdf>.

13. HarvestPlus. The power of orange: Catalyzing delivery of vitamin a maize in Zambia., 2022. Available from: <https://www.harvestplus.org/wp-content/uploads/2022/09/The-Power-of-Orange-Catalyzing-Delivery-of-Vitamin-A-Maize-in-Zambia.pdf>.

14. World Food Programme. WFP logistics cluster. Logistics capacity assessments (lcas). 2022. Available from: <https://dlca.logcluster.org/>.

15. TechnoServe. Zambia large-scale food fortification (LSFF) feasibility assessment, opportunities to expand LSFF in Zambia in collaboration with the food industry. PowerPoint presentation. February 2023.

16. Global Alliance for Improved Nutrition. Gain Zambia food supply data desk review 2023. Unpublished dataset. 2023.

17. USAID. Large-scale food fortification programming guide. Washington, DC: United States Agency for International Development, 2022. Available from: <https://agrilinks.org/post/usaid-large-scale-food-fortification-programming-guide-supporting-food-fortification-country>.

18. Global Fortification Data Exchange. Dashboard: Country fortification. 2023. Available from: <http://www.fortificationdata.org>.

19. Zambia Statistics Agency, Ministry of Health (MOH) Zambia, ICF. Zambia Demographic and Health Survey 2018. Lusaka, Zambia, and Rockville, Maryland, USA: Zambia Statistics Agency, Ministry of Health, and ICF, 2019. Available from: <https://dhsprogram.com/pubs/pdf/SR265/SR265.pdf>.

20. Save the Children UK. The cost of diet tool (cotd). London, UK: Save the Children, 2018.

21. World Health Organization, Food and Agricultural Organization. Vitamin and mineral requirements in human nutrition. Second edition. World Health Organization and Food and Agriculture Organization of the United Nations, 2004.

22. National Food and Nutrition Commission. Zambia food composition tables-4th edition. 2009. Available from: <https://www.nfnc.org.zm/download/zambia-food-composition-tables-4th-edition/>.
